# Genome-Wide Association Data Reveal a Global Map of Genetic Interactions among Protein Complexes

**DOI:** 10.1371/journal.pgen.1000782

**Published:** 2009-12-24

**Authors:** Gregory Hannum, Rohith Srivas, Aude Guénolé, Haico van Attikum, Nevan J. Krogan, Richard M. Karp, Trey Ideker

**Affiliations:** 1 Department of Bioengineering, University of California San Diego, La Jolla, California, United States of America; 2 Department of Toxicogenetics, Leiden University Medical Center, Leiden, The Netherlands; 3 Department of Cellular and Molecular Pharmacology, University of California San Francisco, San Francisco, California, United States of America; 4 California Institute for Quantitative Biosciences, University of California San Francisco, San Francisco, California, United States of America; 5 Department of Electrical Engineering and Computer Science, University of California Berkeley, Berkeley, California, United States of America; 6 California Institute for Quantitative Biosciences, University of California Berkeley, Berkeley, California, United States of America; 7 Department of Medicine, University of California San Diego, La Jolla, California, United States of America; University of Washington, United States of America

## Abstract

This work demonstrates how gene association studies can be analyzed to map a global landscape of genetic interactions among protein complexes and pathways. Despite the immense potential of gene association studies, they have been challenging to analyze because most traits are complex, involving the combined effect of mutations at many different genes. Due to lack of statistical power, only the strongest single markers are typically identified. Here, we present an integrative approach that greatly increases power through marker clustering and projection of marker interactions within and across protein complexes. Applied to a recent gene association study in yeast, this approach identifies 2,023 genetic interactions which map to 208 functional interactions among protein complexes. We show that such interactions are analogous to interactions derived through reverse genetic screens and that they provide coverage in areas not yet tested by reverse genetic analysis. This work has the potential to transform gene association studies, by elevating the analysis from the level of individual markers to global maps of genetic interactions. As proof of principle, we use synthetic genetic screens to confirm numerous novel genetic interactions for the INO80 chromatin remodeling complex.

## Author Summary

One of the most important problems in biology and medicine is to identify the genetic mutations that affect human traits such as blood pressure, longevity, and onset of disease. Currently, large scientific teams are examining the genomes of thousands of people in an attempt to find mutations present only in individuals with certain traits. Until now, mutations have been largely examined in isolation, without regard to how they work together inside the cell. However, large pathway maps are now available which describe in detail the network of genes and proteins that underlies cell function. Here we show how to take advantage of these pathway maps to better identify relevant mutations and to show how these mutations work mechanistically. This basic approach of combining genetic information with known maps of the cell will have wide-ranging applications in understanding and treating disease.

## Introduction

A central challenge in genetics is to understand how interactions among different genetic loci contribute to complex traits [1]–[7]. In model organisms such as yeast, genetic interactions are typically identified using reverse genetic approaches, in which different pairs of genes are systematically knocked out to create a collection of double mutants. Genetic interaction is indicated when the growth rate of the double mutant is slower than expected (e.g., synthetic sickness or lethality) or faster than expected (e.g., suppression) [4],[8],[9]. Rapid screening of such interactions has been made possible through a variety of methods including Synthetic Genetic Array (SGA) analysis [4], diploid Synthetic Lethality Analysis by Microarray (dSLAM) [3], and epistatic miniarray profiles (E-MAP) [1],[2],[10],[11].

In higher eukaryotes such as humans, reverse genetic analysis has not been so straightforward. Complex traits such as body weight or disease onset can be difficult to study in a cell-based assay, and null mutations are expensive to induce in mammals [12]. Instead, interactions amongst loci have been largely mapped from data generated through forward genetic approaches, such as genome-wide linkage [13] or genome-wide association studies (GWAS) [14],[15]. Such methods leverage naturally occurring mutations in the genome to pinpoint loci that have associations, ideally causal associations, with a trait of interest [7].

Mapping pair-wise locus associations has proven remarkably difficult, however. The most basic approach is to perform an exhaustive two-dimensional (2D) scan, in which all pairs of genetic markers are tested for joint association with the phenotype. Because billions of marker pairs must be tested, 2D scans are computationally demanding and suffer from low statistical power due to multiple hypothesis testing. One method to partially mediate this problem is to initiate searches for pair-wise interactions only for markers with strong individual effects [14],[15]. Two recent studies by Storey *et al.* and Litvin *et al.* used this approach while accounting for information shared across multiple traits to further enhance statistical power [16],[17]. These results indicate a major role for genetic interactions in the heritability of complex traits. However, it is likely that the interactions uncovered to date represent only a fraction of the true genetic network.

Here, we show that both the power and interpretation of genetic interactions derived from association studies can be significantly improved through integration with information about the physical architecture of the cell. We apply this integrative approach to an association study conducted in yeast, yielding a genetic network that complements, extends, and validates networks assembled through reverse genetic methods.

## Results

### Bi-clustering of marker pairs defines a network among genomic intervals

We analyzed a recent GWAS in yeast which analyzed a population of 112 segregants resulting from a cross of a laboratory *S. cerevisiae* strain with a wild isolate [5]. For each segregant, the states of 1,211 unique markers (genotypes) were mapped along with the expression profile of 5,727 genes (traits) (Table S1). To identify pairs of markers that genetically interact— i.e. for which the joint state of the marker pair was associated with one or more gene expression traits— we considered the method of Storey *et al*. [17] which provides the best marker pair for each expression trait, resulting in a set of 4,687 distinct marker-marker interactions (removing redundancies due to marker pairs that associate with multiple traits).

**Figure 1:**
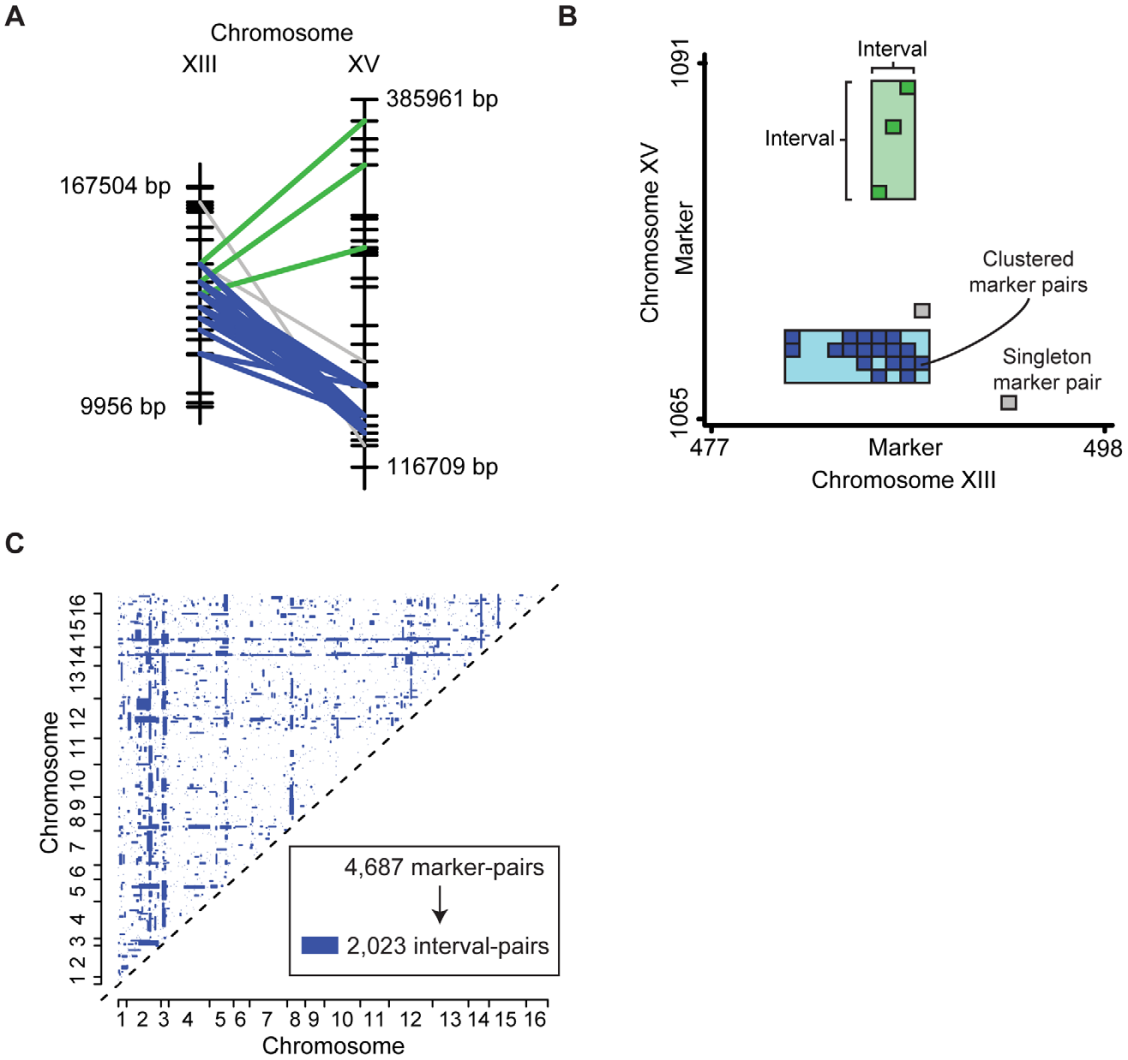
Using genome-wide association data to identify natural genetic interactions. (A) Two interacting interval pairs (green and blue) which represent significantly dense groups of marker-marker interactions are shown. (B) A matrix view of the same genomic regions. The blue and green interval pairs appear as two rectangles. (C) The entire set of marker pairs was bi-clustered to form a set of high-confidence interval pairs (blue rectangles).

A preliminary examination of the genotype data showed few recombinations between neighboring markers, indicating that markers in close proximity were in linkage disequilibrium (LD). As a result, neighboring markers were often found to display similar patterns of interactions (Figure 1A). In much the same way that LD has allowed neighboring markers to be grouped into haplotype blocks [18], we reasoned that LD between neighboring markers could also be exploited to enhance marker-marker interactions. To this end, we developed a bi-clustering algorithm to identify groups of marker-marker interactions that fall across common genomic intervals (Figure 1B; see Methods). We reasoned that bi-clustering the marker pairs might provide two distinct advantages: First, it allows many statistically insignificant marker-marker interactions to reinforce a single interval-interval interaction. Second, it leverages the structure between neighboring marker pairs to identify with greater precision the interval of DNA underlying the variance in a given trait.

Applied to the marker pairs from Storey *et al.*, the bi-clustering procedure yielded a network of 2,023 interactions between 1,977 genomic intervals (Figure 1C). Of these, 695 interval pairs garnered support from multiple marker pairs (five on average). The remaining 1,328 interval pairs consisted of singleton marker-marker interactions, which were not found to cluster with any others. The complete network of interval-interval interactions can be found in Table S2. We refer to this network as a natural genetic network since it is derived from natural rather than engineered mutations.

### Natural interactions define a map of functional links between protein complexes

A common interpretation of genetic interactions measured in reverse genetic screens has been the “between-complex” or “between-pathway” model, in which interactions are found to span pairs of protein complexes or functional annotations. Such complex-complex interactions have been instrumental in identifying synergistic or compensatory relationships [4],[8],[19]. Similarly, pairs of functional terms have served to identify functions that are cooperative or buffer one another [4].

To evaluate natural networks in this fashion, we examined all pairs of documented protein complexes (out of 302 in Gavin *et al*. [20] or the Munich Information Center for Protein Sequences [MIPS] [21]) and all pairs of functional terms (out of 1,954 terms in the Gene Ontology [GO] [22]) for enrichment for natural genetic interactions. As further described in Methods, we inspected all complex pairs and found 208 significant interactions in the natural network (False Discovery Rate<5%; Table 1). Similarly, we identified 17,714 significant interactions between functional terms. In contrast, far fewer results were found for complex or term interactions derived from the raw marker pairs of Storey *et al*. prior to bi-clustering these data into intervals (Table 1). The full set of complex-complex and term-term interactions are available as a resource in Table S3 and on http://www.cellcircuits.org/qtlnet/.

**Table 1:** Correspondence of interval and marker pairs with complexes and functions.

				Between		Within	
		Nodes†	Edges‡	Complexes	Terms	Complexes	Terms
**Storey ** ***et al.***							
	Bi-clustering*	1,977	2,023	208	17,714	0	12
	Raw Marker Pairs	1,157	4,687	38	3,546	0	3
**Full 2D ANOVA scan** **							
	Bi-clustering	1,387	964	0	19	0	0
	Raw Marker Pairs	1,141	4,687	0	0	0	0
**Synthetic Genetic Analysis**		2,117	29,275	140	1,833	13	33

**†:** Node definition: For Storey *et al.* and Full 2D ANOVA, nodes represent genomic intervals. For the synthetic network, nodes represent genes.

**‡:** All cases report the number of distinct interactions in the network, removing redundancies due to marker pairs that associate with multiple traits (Storey *et al.*, Full 2D ANOVA) or gene pairs scoring positive in multiple data sets (Synthetic Genetic Analysis).

*: These bi-clustered interval pairs were used to define the “Natural Network” explored in this work.

**: We also considered an exhaustive scan of all marker pairs using two-way analysis of variance (ANOVA). The most significant 4,687 marker-marker interactions (Table S7) were taken to match the number of interactions from Storey *et al.* (Text S1). Both the raw marker-pairs and the bi-clustered interval network identified substantially fewer enrichments than the Storey *et al.* method.

**Figure 2:**
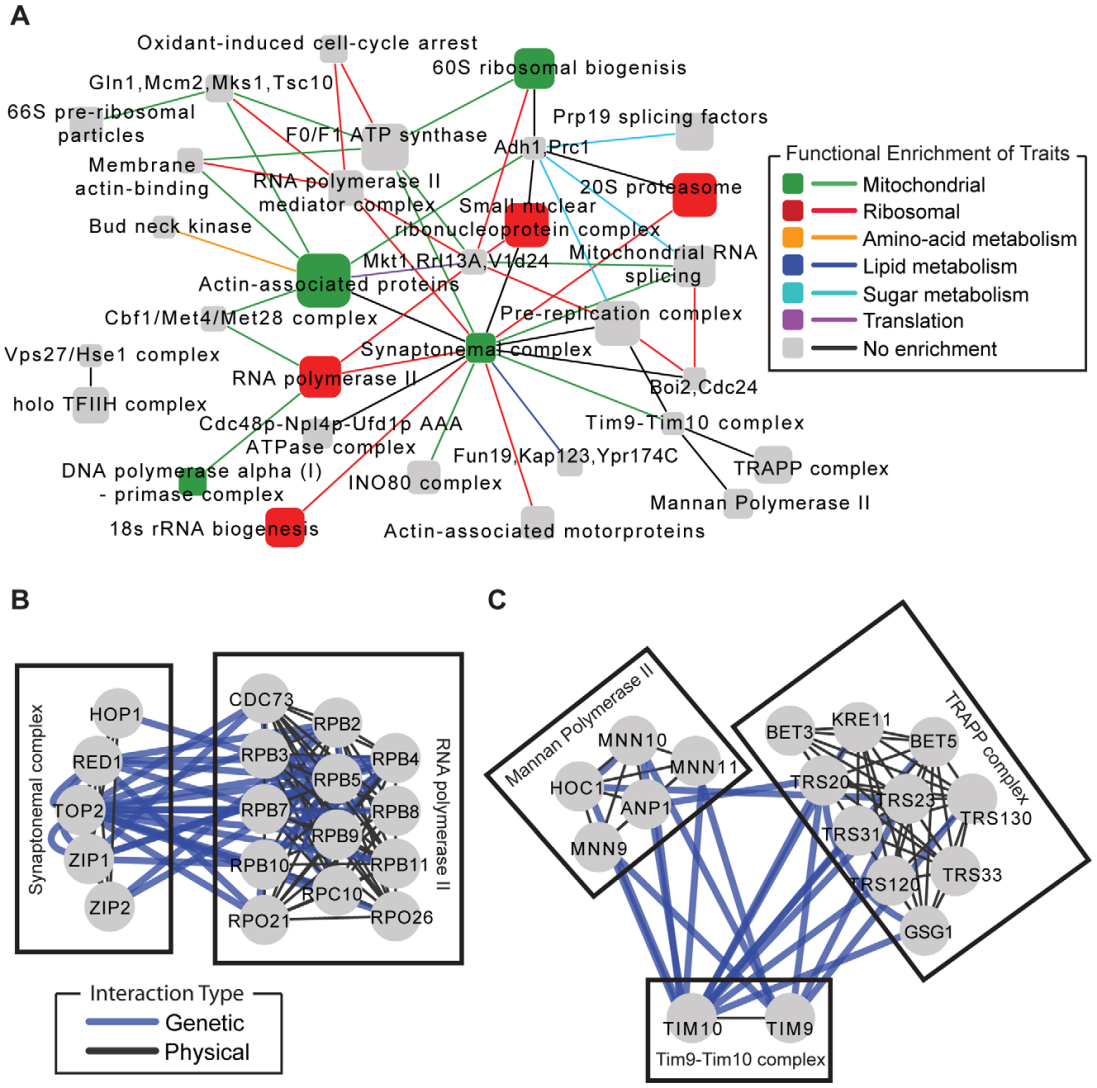
Natural genetic networks elucidate pathway architecture. (A) A global map of the top 50 complex–complex interactions found using the natural network. Each node represents a protein complex and each interaction represents a significant number of genetic interactions (False Discovery Rate<5%) [49]. We analyzed the set of gene expression traits associated with each complex-complex interaction for functional enrichment using the hypergeometric test. Nodes and edges are colored according to the functional enrichment of gene expression traits underlying the natural interactions (Bonferroni P′<0.05). Node sizes are proportional to the number of proteins in the complex. When available, nodes have been labeled with the common name of the complex. (B,C) Two specific examples of complexes spanned by dense bundles of natural genetic interactions.

Figure 2A shows a map of the 50 most significant complex-complex interactions. Because gene expression is the phenotypic trait, each complex-complex interaction is linked to a cluster of gene expression levels that it regulates (with each cluster containing an average of 287 genes). As the map integrates many traits simultaneously, it is distinct from previously-published genetic networks which have relied on cell viability as the single readout of interest. We found that two-thirds of the complex-complex interactions were linked to gene expression clusters that were highly functionally coherent (Figure 2A). In contrast, less than one one-hundredth of interval-pairs were found to influence a set of genes belonging to a single pathway or function. Thus, we conclude that integration of epistatic interactions with protein complex maps helps to filter spurious interactions while simultaneously providing a putative mechanism for the pair-wise associations.

As an illustrative example, Figure 2B shows the natural genetic interactions supporting a functional link between the synaptonemal complex and RNA Polymerase II. Mutations in the *TOP2* gene of the synaptonemal complex have been shown to lead to higher levels of mitotic recombination in rDNA which can result in amplification and deletion of the rDNA array [23]. RNA polymerase II is responsible for the transcription of small nucleolar RNAs (snoRNAs) that physically and functionally interact with many other proteins required for ribosomal biogenesis [24]. Indeed, we found that the gene expression traits linked to this interaction were enriched for ribonucleoprotein complex biogenesis and ribosome biogenesis (both P′ = 10^−8^ by hypergeometric test; P′ is a Bonferroni corrected p-value).

Figure 2C centers on two of the interactions for the Tim9-Tim10 complex, an essential component of the TIM machinery responsible for the transport of carrier proteins from the cytoplasm to the inner mitochondrial membrane [25]. Tim9-Tim10 is genetically connected with Mannan Polymerase II and the TRAPP complex. Mannan Polymerase II is a component of the secretory pathway and is involved in lengthening the mannan backbone of cell wall and periplasmic proteins [26]; the TRAPP complex plays an important role in trafficking of proteins from the golgi to the cell periphery [27]. The abundant genetic interactions between Tim9-Tim10 and these two complexes suggest they may jointly influence the make-up of cell surface proteins, possibly through control of trafficking. Consistent with this hypothesis, disruption of mitochondrial function has been shown to influence cell wall composition, including levels of phosphopeptidomannans [28].

For comparison to the between-complex model, we also examined the natural genetic network for support for a “within-complex” model, in which single functional terms or complexes are enriched for genetic interactions among their member genes [4],[8],[19]. Searching across the 1,954 GO terms and 302 complexes, the natural network identified only 12 enriched GO terms and no significant complexes (Table 1 and Table S3). Thus, genetic interactions in naturally-derived networks are far less likely to occur within a single pathway than to span between pathways. This result mirrors what has been observed in analysis of reverse genetic interaction networks, particularly amongst interactions characterized as synthetic lethal or synthetic sick, which have been shown to interconnect different pathways that are functionally synergistic or redundant [19],[29].

### Complementarity between natural and synthetic genetic networks

Next, we asked whether the natural genetic network had any direct overlap with “synthetic” networks derived using reverse genetic approaches such as SGA, dSLAM, or E-MAP platforms. To address this question, we considered four synthetic interaction networks: a network by Tong et al. [4] including comprehensive interaction screens for 132 genes using SGA, a genetic network governing DNA integrity identified using dSLAM [3], and E-MAPs centered on chromosomal biology [2] and RNA processing [1]. The combined network from these four sources consisted of 2,117 genes linked by 29,275 genetic interactions. As with the natural network, we confirmed that interactions in the combined synthetic network were more likely to fall between functional terms and protein complexes than within them (Table 1 and Table S4).

**Figure 3:**
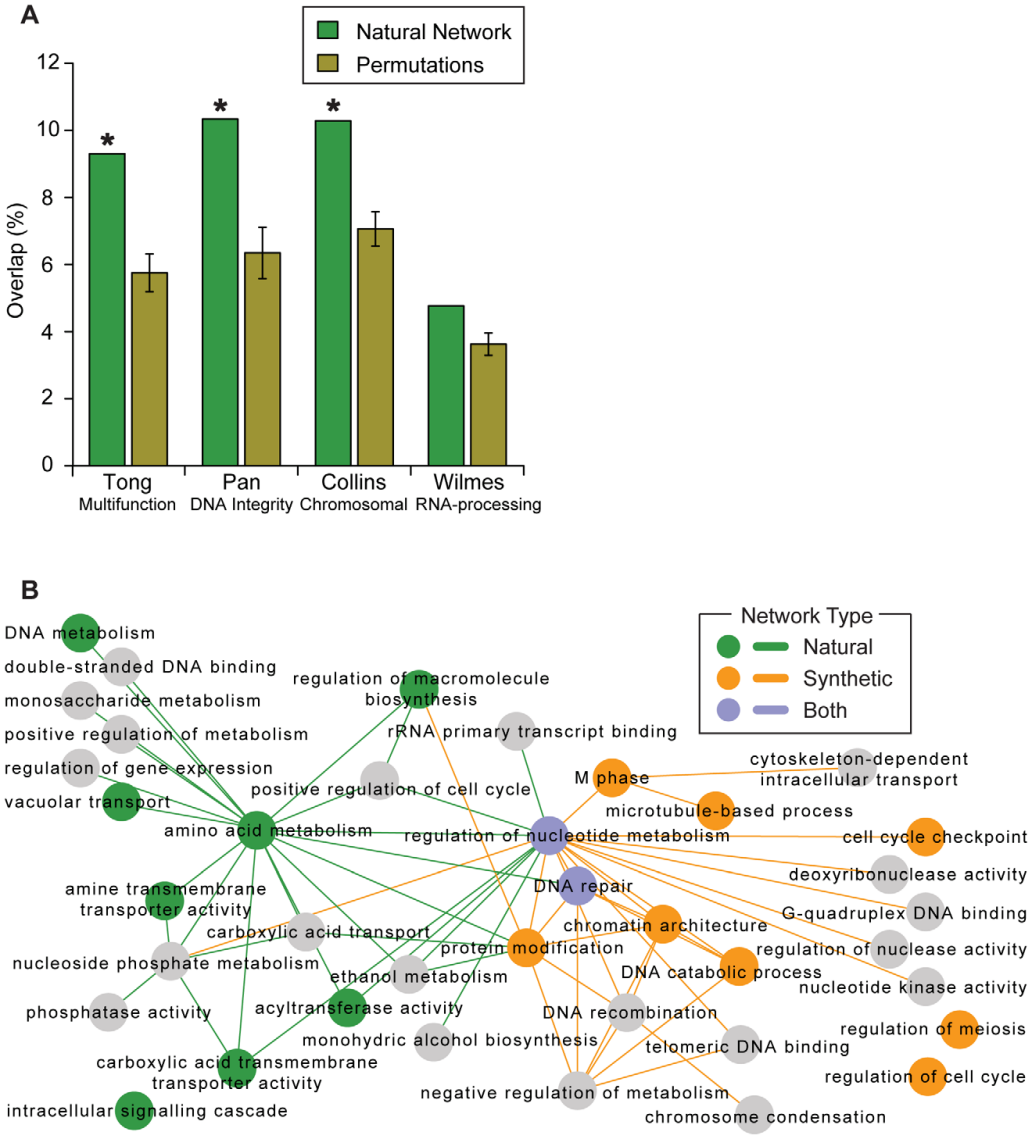
Comparison of the natural and synthetic networks. (A) The overlap between the natural network and four previously-published synthetic genetic networks (Tong [4], Pan [3], Collins [2], Wilmes [1]) is shown as a percentage of the synthetic network size. An asterisk indicates significance at P<0.05. (B) A map of the functions and functional relationships supported by either the natural or synthetic networks. Each node represents a broad GO term, with colors (green, orange, blue) indicating terms that contain many within-term interactions (Text S1). Edges show the top 30 between-term interactions for each of the natural and synthetic networks. Two broad GO terms (regulation of nucleotide metabolism and DNA repair) contained many within-term interactions in both the natural and synthetic networks.

To evaluate overlap, an interaction in the synthetic network was considered “supported” if the two genes mapped into two different intervals that were found to interact in the natural network. As shown in Figure 3A, the natural network supported on average 8.7% of interactions across the four synthetic networks as opposed to 5.7±0.5% expected by chance (Text S1). Thus, some regions are shared in common between natural and synthetic networks, although these regions appear to represent a minority of all genetic interactions.

**Figure 4:**
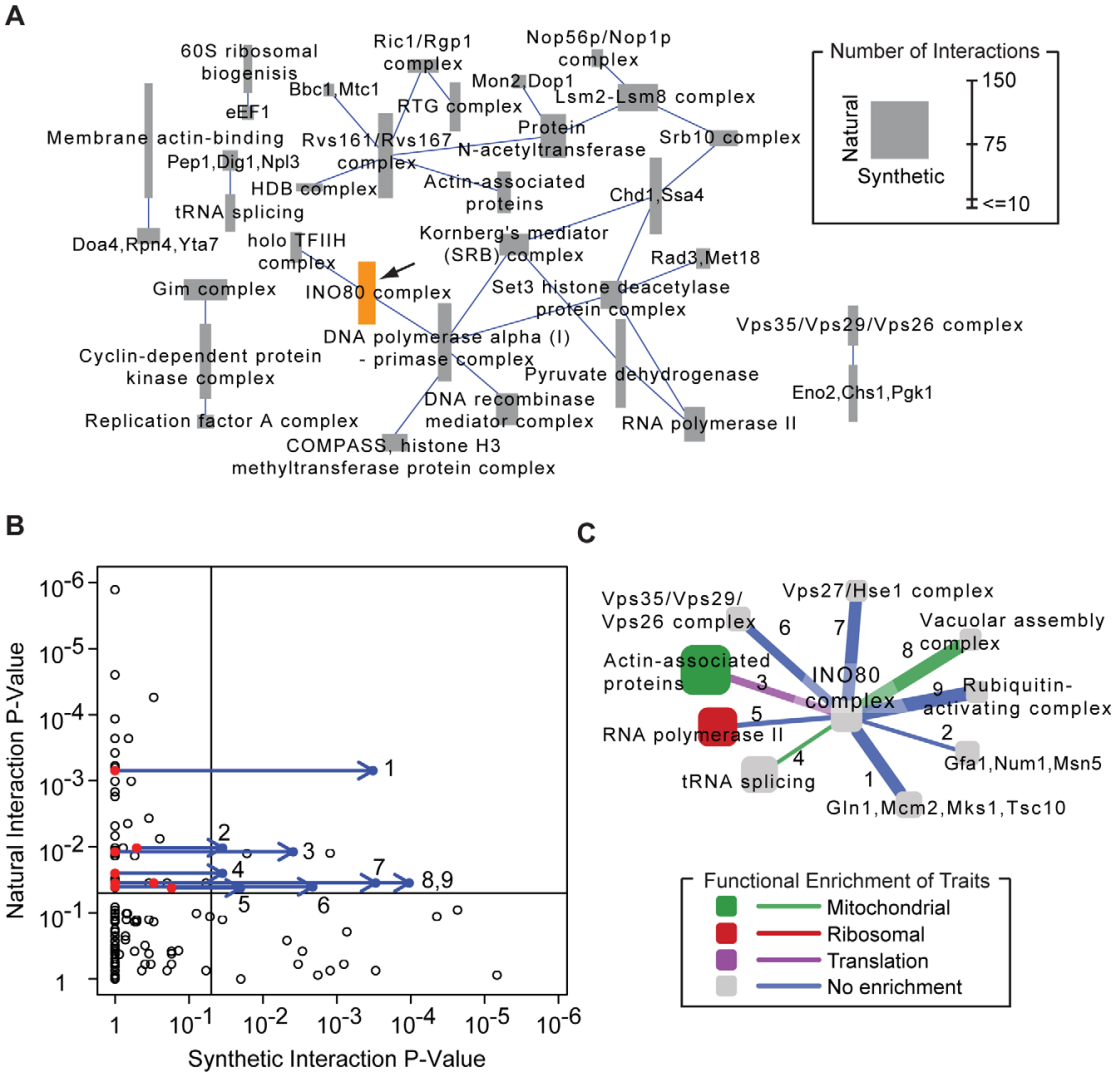
Guiding synthetic genetic screens using natural genetic networks. (A) Complex-complex interactions common to both the natural and synthetic networks at a relaxed threshold of P<0.05. Many of these complexes, including INO80 (orange), have more coverage in the natural network (node height) than in the synthetic network (node width). (B) Each point in the scatter plot represents the significance of support for a possible complex-complex interaction with INO80 from the natural (y-axis) versus synthetic (x-axis) networks. Due to low coverage, comparatively few complex pairs have support in the synthetic network. New E-MAP data for INO80 support nine new complex-complex interactions predicted by the natural network (blue arrows). (C) A network of natural genetic interactions for INO80 validated by the new E-MAP. Functional enrichment for traits is shown as in Figure 2. The thickness of each link is proportional to its support in the new genetic interaction screen.

We found that these common genetic interactions took place among genes encoding basal transcriptional activators (“regulation of nucleotide metabolism”, Figure 3B) including components of RNA polymerase II, Kornberg's mediator complex, the holo TFIIH complex, INO80, SET3, and COMPASS (Figure 4A). The expression traits linked to these common interactions were for genes encoding the cytosolic ribosome (P′<10^−47^), cell cycle checkpoints (P′<10^−15^, including *RAD9* and *DDC1*), and mitochondrial electron transport (P′<10^−12^). Thus, interactions that overlap between natural and synthetic genetic networks take place largely among core transcriptional activators and influence expression of core metabolic processes.

### Novel interactions of the INO80 complex as suggested by natural networks

One prominent complex highlighted by both natural and synthetic interactions was INO80, a multi-subunit ATP-dependent chromatin remodeling complex (Figure 4A). At its core is the Ino80 protein, an ATPase of the SNF2 family which functions as the catalytic subunit. Recent studies have demonstrated that INO80 chromatin remodeling activity contributes to a wide variety of pivotal processes, including transcription, DNA replication, and DNA repair [30]–[33]. Consistent with these processes, both the natural and synthetic networks supported interactions of INO80 with TFIIH and alpha(I)-primase. However, INO80 had far more interactions in the natural network than the synthetic one. This result is reflected in Figure 4A (large height versus width of the INO80 node) and more explicitly in Figure 4B, which plots the p-values in the natural versus synthetic network for all complex pairs involving INO80. This plot suggests that the reason for few synthetic interactions is lack of coverage: most complex pairs (82%) have simply not yet been tested for interaction using reverse genetic screens, placing them at a significance score of P = 1 (i.e., on the y-axis of Figure 4B).

To fill this gap, we genetically analyzed three genes encoding members of the INO80 complex (Arp8, Ies3, Nhp10) using the quantitative E-MAP approach. Complete genomic deletions of each gene were screened against a standard array of 1,536 mutants to select double mutant combinations whose growth rates were slower or faster than expected (Methods). This screen uncovered 496 novel genetic interactions (Table S5) supporting 20 complex-complex relationships (P<0.05; Table S6). Nine of the complex-complex interactions were also supported by the natural network, including interactions with four complexes (tRNA splicing, RNA polymerase II, Actin-associated proteins, and the Vps35/Vps29/Vps26 complex) that were already present in the common complex interaction map (see Figure 4B and 4C).

The relationships identified here implicate a number of novel links between INO80-mediated chromatin remodeling and a wide range of important cellular processes. For example, numerous genetic interactions were identified between INO80 and RNA Polymerase II. There is substantial evidence demonstrating that the rate of transcriptional elongation by RNA Polymerase II is reduced in the presence of nucleosomes and requires chromatin-modifying activities [34]. Since INO80 has been shown to mobilize/remove nucleosomes [32],[35], this functional link may indicate that the two complexes co-operate: INO80 may exchange histones at a particular location to facilitate transcriptional elongation by RNA polymerase II. Indeed, while this manuscript was in review, a new report has implicated a role for INO80 in histone redeposition during RNA polymerase II-mediated transcription of stress-induced genes [36].

Four of the nine novel INO80 interactions are involved in various aspects of vacuolar protein degradation including transport of hydrolases to the vacuole (Vps35/Vps29/Vps26 complex and Vps27/Hse1 complex), vacuole biogenesis (Vacuolar assembly complex), and targeting of proteins for degradation (Rubiquitin-activating complex). Given INO80's role in transcription [32], the new interactions suggest that these complexes work in tandem to regulate the expression level of certain proteins, with INO80 controlling the level of transcription and these four complexes controlling the rate of protein degradation. This work serves as an example of how the broad coverage in the natural network can be used to focus future genetic screens and provide the basis for many mechanistic follow-up studies.

## Discussion

Currently, mapping genetic interactions using GWAS faces two major challenges: a lack of statistical power for finding genotype-phenotype associations, and a lack of tools for understanding the molecular mechanisms behind the associations found to be significant [14],[15],[37]. In this study, we have demonstrated that such challenges can be partly overcome by (1) accounting for bi-cluster structure in the data and (2) by integrating genetic interactions derived from GWAS with protein complexes and functional annotations. The result is a map of protein complexes and pathways interconnected by dense bundles of genetic interactions, which raises statistical power and provides biological context to the genetic interactions uncovered in natural populations.

Despite exhibiting some overlap (8.7%), there was also much divergence between the natural and synthetic networks. Such divergence might be explained by a number of factors. First, the two types of genetic networks have major differences with respect to coverage and power. Natural networks are based on genome-wide variations and thus nearly all gene pairs are tested for pairwise interaction— i.e., the coverage of gene pairs is practically complete. This large coverage comes at the price of low statistical power: gene association studies are limited by the number of individuals that can be surveyed which, in turn, limits the power of natural genetics to detect any given genetic interaction. On the other hand, a reverse genetic interaction screen explicitly tests the growth rate of gene pairs, with high power to detect interaction. However, the set of gene pairs that can be tested in a single study is limited by the throughput of the screening technology. The synthetic genetic network used here was a combination of four such studies which collectively cover approximately 5% of yeast gene pairs. Future efforts may seek to complement the coverage of reverse genetic screens by using natural genetics, or to improve the power of gene association studies through focused reverse genetic analysis. Here, we have demonstrated this concept by expanding the coverage of the synthetic network around the INO80 complex, based on the conserved interactions we found for this complex in both types of networks.

Even with equivalent coverage and power, the two types of network would still likely diverge due to their different means of perturbation. The natural network is driven by variations in genome sequence including SNPs, repeat expansions, copy number variations, and chromosomal rearrangements which lead to a variety of effects on gene function such as hypo- and hypermorphic alleles, null alleles, and so on. In contrast, synthetic networks predominantly consist of complete gene deletion events, which are rarely experienced in nature and lead exclusively to null alleles.

A final difference is phenotype— the natural and synthetic networks in this study differ markedly in the underlying phenotypic traits they have measured, relating to gene expression versus cell growth, respectively. It is important to note, however, that the differences in traits are specific to the currently available data sets. They are not inherent to either mapping approach, and in general one can imagine synthetic genetic interactions related to gene expression (see Jonikas *et al*. for a recent example [38]) or natural interactions related to a single phenotypic trait such as cell viability or disease (which in fact describes the majority of GWAS data generated to-date for humans) [7].

Despite all of these differences, we did observe a significant number of natural and synthetic genetic interactions in common. It is tempting to speculate that these common interactions might share certain characteristics with regard to cellular function. In particular, we found that natural interactions also present in the synthetic network were linked to expression levels of ribosomal genes as well as to core components of respiration and cell cycle. Several studies have noted a correlation between the expression levels of ribosomal or mitochondrial genes and growth rate [39],[40]. Thus, the overlap between natural and synthetic interactions seems to occur among genes that strongly influence expression traits related to growth.

A common issue in association studies, known as the “fine mapping problem” [41],[42], is that a strongly associated marker will fall near many candidate genes, leaving it ambiguous as to which of these candidates is the causal factor. Numerous methods have been developed to refine or prioritize these candidates, often through incorporation of orthogonal information [43]. An extension of this problem applies to marker-marker interactions, which typically implicate one of many possible pairs of genes. Here, we have mitigated this problem by summarizing markers into protein complexes and functional terms. However, ambiguities can still arise in cases where several complex-complex interactions are supported by the same underlying set of marker pairs. Since it is likely that only one of these interactions is causally linked to phenotype, further work may be necessary to prioritize these candidates. It is important to note, however, that fine-mapping issues will be less of a concern in humans than in yeast, given the higher density of available markers which will improve the resolution in identifying causal genes.

In summary, we have demonstrated that the logical framework developed for analysis of synthetic genetic networks can also be readily applied to natural genetic networks. Biologically and clinically, the clear and immediate application is towards the analysis of genome-wide association studies in humans. Many diseases, both common and rare, have so far been opaque to genome-wide association analysis [44]. The key question will be whether, using integrative maps such as those developed here, they can become less so.

## Methods

### Marker pair bi-clustering

An interval is defined as a set of one or more contiguous markers along the chromosome. A pair of intervals induces a set of *m* tested marker pairs of which *k* pairs are found to interact, drawn from a total genome-wide pool of *N* tested marker pairs of which n are found to interact. An exhaustive genome-wide scan is performed to identify interacting interval pairs, i.e. those that are enriched for marker-marker interactions, as follows. The counts (*m*, *k*) are tallied for all possible pairs of intervals (up to a maximum of 60 markers per interval) using a recursive algorithm in which the entire space of marker pairs is represented as an upper-triangular matrix A with each row and column denoting a marker. An interval pair is represented by a submatrix Ai,j,a,b, where *i,j* are the starting row and column indices and *a,b* are the dimensions of the submatrix. The number *k*i,j,a,b of interacting marker pairs in a submatrix is determined using the formula:
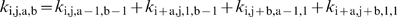



An identical formula is used to count the number of tested marker pairs in each interval pair (substitute *m* for *k*). Following computation of the (*m*, *k*) counts, every interval pair is assigned a p-value of enrichment for marker-marker interactions based on the four parameters *m*, *k*, *N*, *n* using the hypergeometric distribution. The natural network is then assembled in an iterative fashion, where the most significant interval pair is selected from among all possible interval pairs, after which all interval pairs which contain any overlapping marker pairs (interacting or non-interacting) are removed from consideration. The process is repeated until there are no interval pairs remaining, which ensures that the final set of interval-interval interactions comprising the natural network is disjoint.

### Comparison of bi-clustering to a naïve algorithm

We considered that the improved performance of bi-clustering might be non-specific, i.e., that simpler methods for expanding marker-marker pairs to form genomic intervals might perform equally well. As one possibility, we compared the bi-clustering approach to a naïve algorithm for generating interval-interval interactions, in which raw marker pairs were expanded to encompass the nearest *x* neighboring markers on either side. However, as shown in Figure S1 this naïve expansion method performed substantially worse than bi-clustering at identifying term-term or complex-complex interactions, for any choice of *x*, suggesting that bi-clustering identifies more appropriate interval boundaries for each natural genetic interaction.

### Mapping genes to intervals

The chromosomal coordinates of open reading frames (ORFs) for all yeast genes were obtained from the *Saccharomyces* Genome Database [45]. Each gene was assigned to all markers found within its ORF and to the nearest marker within a window of *x* = 100 kb on either side (Figure S2). This mapping procedure resulted in a discrete number of genes mapped to a given marker. Intervals were mapped to all genes assigned to their constituent markers, again resulting in a discrete number of genes mapped to an interval.

The complex-complex interactions identified in the natural network were robust to the particular choice of window size *x*. We varied *x* over a range of distance thresholds from 0 to 100 kb. As shown in Figure S3, the resulting complex-complex interactions implicated by the natural network had a high degree of overlap with the results obtained using the original mapping procedure.

### Enrichments of interactions within and between complexes and terms

A within-complex (within-term) model is defined as the set of all gene pairs falling within a given physical complex (functional GO term). A between-complex (between-term) model is defined as the set of all gene pairs that span two complexes (terms), such that one gene belongs to the first complex, the other gene belongs to the second complex, and neither gene belongs to both. For each model we compute *k*, the number of gene pairs “supported” (see main text) by the network. The significance of this support is assessed using the hypergeometric distribution, governed by *k* and three additional parameters:

*n*. The total number of gene pairs induced by the model.

*m*. The total number of gene pairs having support in the entire network.

N. The total number of gene pairs in the tested space of the entire network.

Counts for all four parameters are based only on pairs of genes found in the corresponding space of interactions tested by the network and covered by the given annotation set (complexes or terms). Further details are given in Text S1. All models are visualized using Cytoscape [46].

### Removing the effects of non-random gene order on annotation enrichment

The above enrichment tests assume independence of genetic interactions from protein complexes and functional terms. However, intervals in the natural network typically cover several consecutive genes, which are more likely to be of similar function than genes chosen at random [47]. To correct for this effect, each complex/term annotation is assigned a score *P_min_*∈[0, 1] measuring the degree to which its member genes are clustered [*P_min_* → 0] versus dispersed [*P_min_* → 1] along the genome (see Text S1 for more details). Annotations with *P_min_*<*p*_T_ are removed from further consideration. We use a stringent threshold of *p*_T_ = 0.1 for physical complexes and *p*_T_ = 0.3 for functional terms resulting in less than one erroneous complex-complex or term-term interaction identified in randomized networks (Figure S4 and Figure S5). Further details regarding the randomization procedure is provided in Text S1. A list of the complexes used in this study is provided in Table S8.

### INO80 Epistatic Mini-Array Profile (E-MAP)

The *arp8Δ*, *nhp10Δ*, and *ies3Δ* knockout strains were constructed and E-MAP experiments were performed as described previously [48]. The array used to generate the double-knockout strains contained 1,536 strains involved in chromatin metabolism (including chromatin remodeling, repair, replication, and transcription) as well as global cellular processes like protein trafficking and mitochondrial metabolism (see Table S5). Genetic interaction scores were computed as described previously [9].

## Supporting Information

Figure S1 Comparison of the bi-clustering method to a naïve approach. A naïve approach for identifying interval-interval interactions was compared to the bi-clustering approach. In the naïve approach, markers involved in a marker-marker interaction were expanded to encompass the nearest k neighboring markers on either side. The naïve approach identified substantially fewer between-pathway enrichments.
(0.14 MB TIF)

Figure S2 Interval to gene mapping. Each gene (diamond) was assigned to all markers (vertical bars) found within its ORF and to the nearest marker within a window of x = 100 kb on either side. Each interval (green bar) inherited the mapping of all constituent markers.
(0.71 MB TIF)

Figure S3 Sensitivity of pathway identification to marker-gene mapping threshold. Genes were mapped to their nearest marker within 100 kbp. We varied this threshold from 0 kbp to 100 kbp to determine what effect it would have on the resulting complex-complex interactions. Overlap of the resulting complex-complex interactions with the results in the manuscript are shown as a Jaccard score.
(0.26 MB TIF)

Figure S4 Choosing a colocalization threshold. The number of interactions identified from permuted natural networks were examined at several colocalization thresholds. Thresholds were chosen which resulted in fewer than one interaction in a typical permuted network (blue arrows).
(0.36 MB TIF)

Figure S5 Additional permutation methods for pathway validation. The number of complex-complex interactions identified by the natural network (dotted line) is compared to the average number of complex-complex interactions identified across 100 permuted interval networks generated using three different procedures. Complex-complex interactions were mapped using either all complexes (unfiltered) or only those with a co-localization p-value above 0.1 (filtered). Error bars indicate one standard deviation.
(0.29 MB TIF)

Table S1 List of genetic markers used in the association study and their genomic locations. A list of the genetic markers and their corresponding genomic locations used in the Brem *et al*. study [5].
(0.09 MB XLS)

Table S2 List of interval-interval interactions in the natural network. A list of interval-interval interactions identified by the bi-clustering algorithm.
(0.20 MB XLS)

Table S3 Significant pathways identified by the natural network. (A) List of significant complex-complex interactions identified. (B) List of significant term-term interactions identified. (C) List of complexes containing a significant number of natural interactions. (D) List of functional terms containing a significant number of natural interactions.
(1.35 MB XLS)

Table S4 Significant pathways identified by the pooled synthetic network. (A) List of significant complex-complex interactions identified. (B) List of significant term-term interactions identified. (C) List of complexes enriched for synthetic interactions. (D) List of functional terms enriched for synthetic interactions.
(0.17 MB XLS)

Table S5 Results of the INO80 E-MAP screen.
(0.31 MB XLS)

Table S6 Novel complex-complex interactions identified in the INO80 E-MAP screen. List of significant complex-complex interactions identified by the new synthetic genetic interactions uncovered in the new E-MAP screen.
(0.02 MB XLS)

Table S7 Results from the exhaustive 2D scan. (A) For comparison with the Storey *et al*. approach [24], the association data were analyzed using a simple 2-way ANOVA (see Text S1). Marker-marker interactions with P<0.18 are presented here. (B) The marker-marker interactions from (A) were bi-clustered to identify 964 interval-interval interactions. Both the raw marker pairs and the interval pairs identified substantially less pathways than the Storey *et al*. approach (Table 1).
(0.69 MB XLS)

Table S8 List of physical complexes used in this study.
(0.04 MB XLS)

Text S1 Supplementary methods.
(0.06 MB DOC)
