# Supplementary figures and images for: Genome-Wide Association Data Reveal a Global Map of Genetic Interactions among Protein Complexes
Source: PLoS Genet. 2009 Dec 24;5(12):e1000782. doi: 10.1371/journal.pgen.1000782 (PMC2788232; doi:10.1371/journal.pgen.1000782)

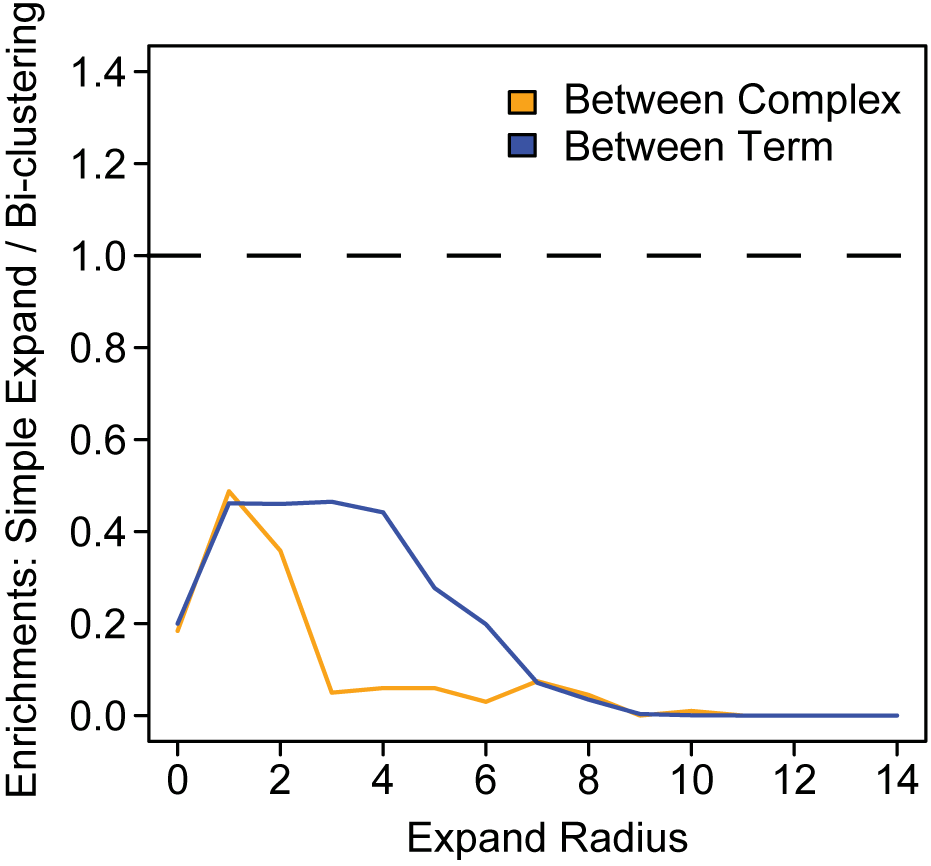

Supplement: Figure S1 — Comparison of the bi-clustering method to a naïve approach. A naïve approach for identifying interval-interval interactions was compared to the bi-clustering approach. In the naïve approach, markers involved in a marker-marker interaction were expanded to encompass the nearest k neighboring markers on either side. The naïve approach identified substantially fewer between-pathway enrichments. (0.14 MB TIF) [file pgen.1000782.s001.tif]

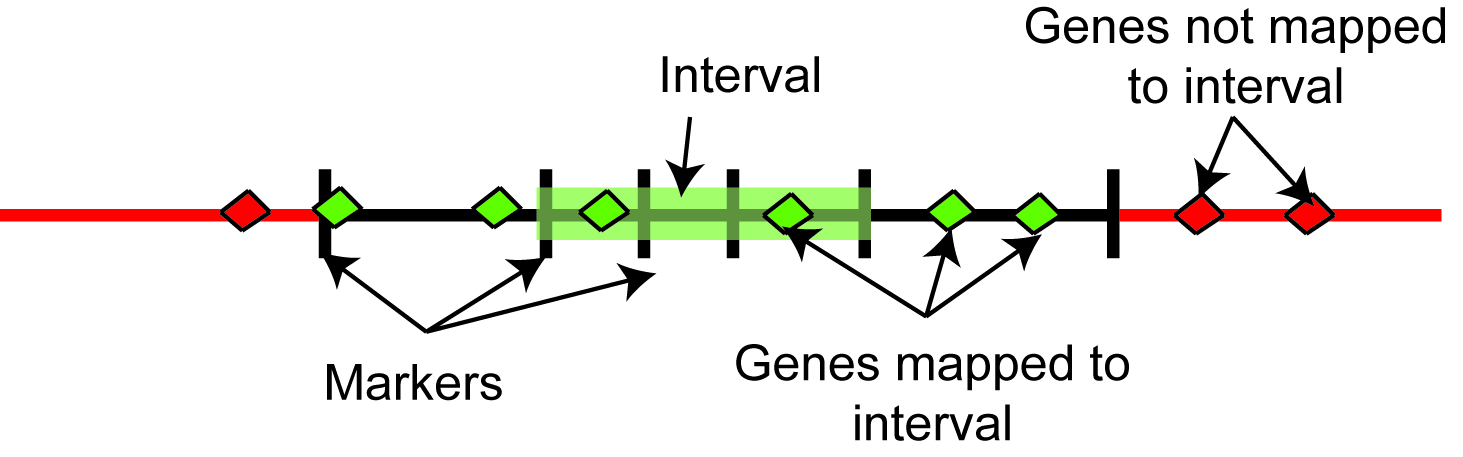

Supplement: Figure S2 — Interval to gene mapping. Each gene (diamond) was assigned to all markers (vertical bars) found within its ORF and to the nearest marker within a window of x = 100 kb on either side. Each interval (green bar) inherited the mapping of all constituent markers. (0.71 MB TIF) [file pgen.1000782.s002.tif]

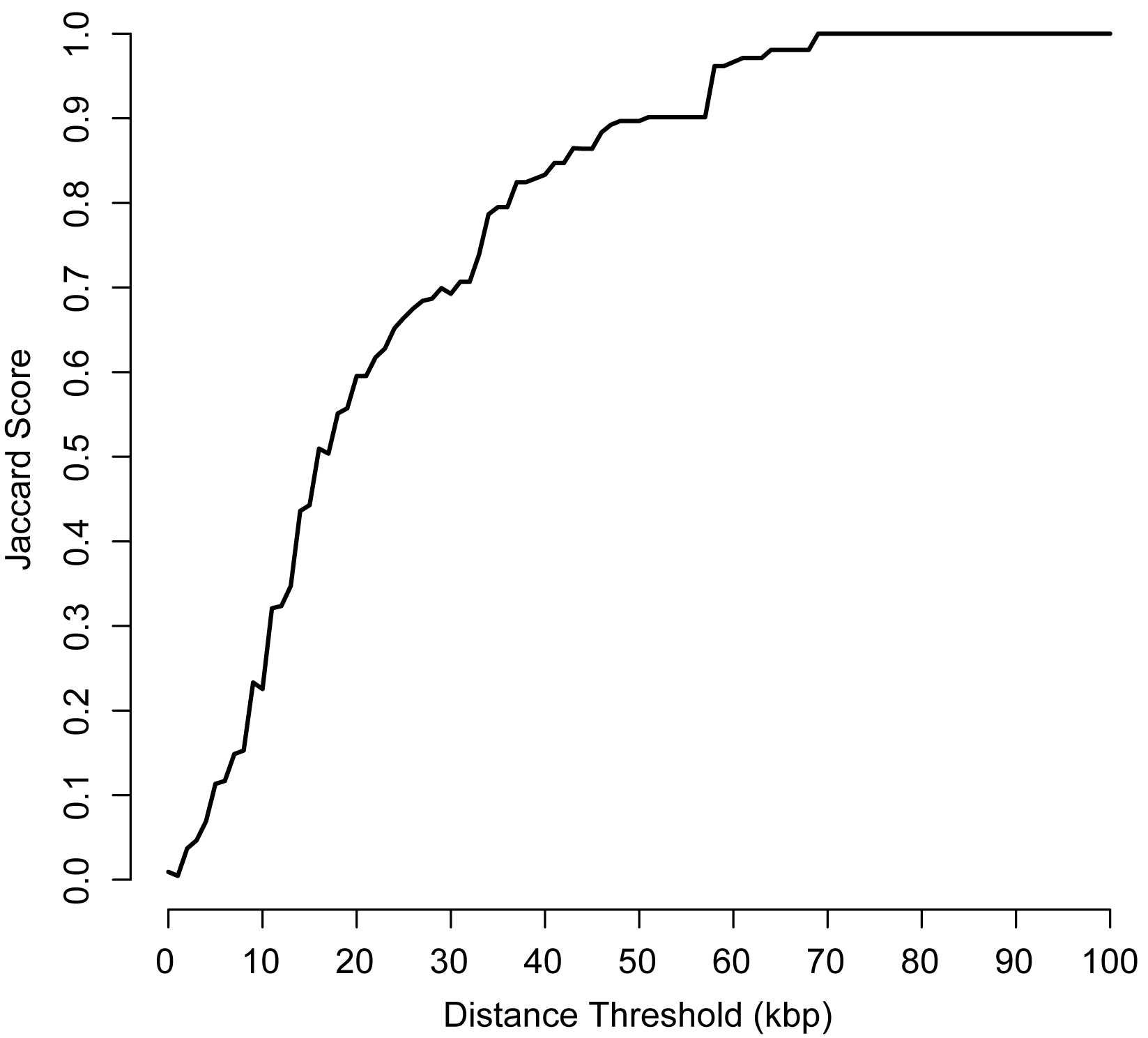

Supplement: Figure S3 — Sensitivity of pathway identification to marker-gene mapping threshold. Genes were mapped to their nearest marker within 100 kbp. We varied this threshold from 0 kbp to 100 kbp to determine what effect it would have on the resulting complex-complex interactions. Overlap of the resulting complex-complex interactions with the results in the manuscript are shown as a Jaccard score. (0.26 MB TIF) [file pgen.1000782.s003.tif]

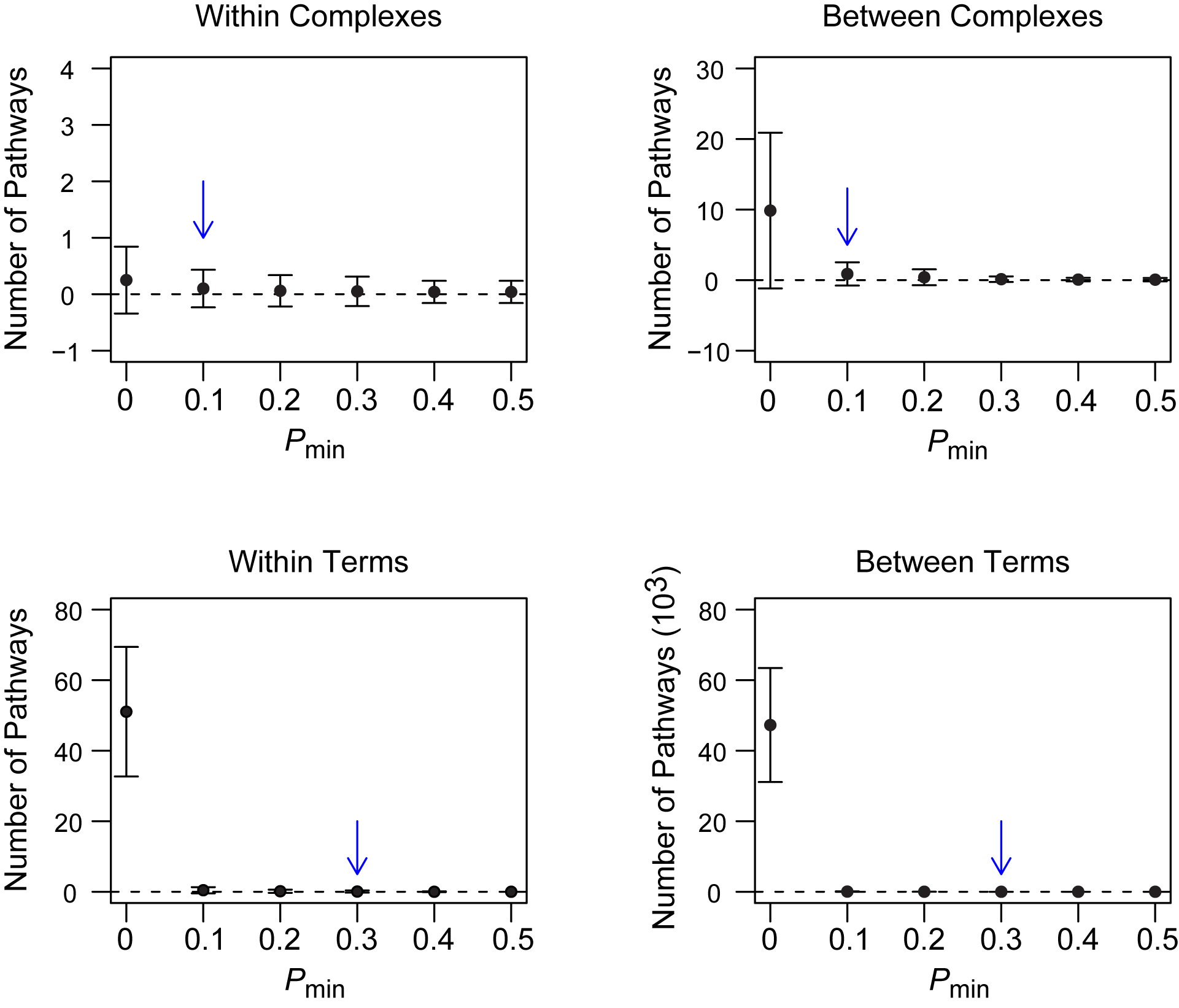

Supplement: Figure S4 — Choosing a colocalization threshold. The number of interactions identified from permuted natural networks were examined at several colocalization thresholds. Thresholds were chosen which resulted in fewer than one interaction in a typical permuted network (blue arrows). (0.36 MB TIF) [file pgen.1000782.s004.tif]

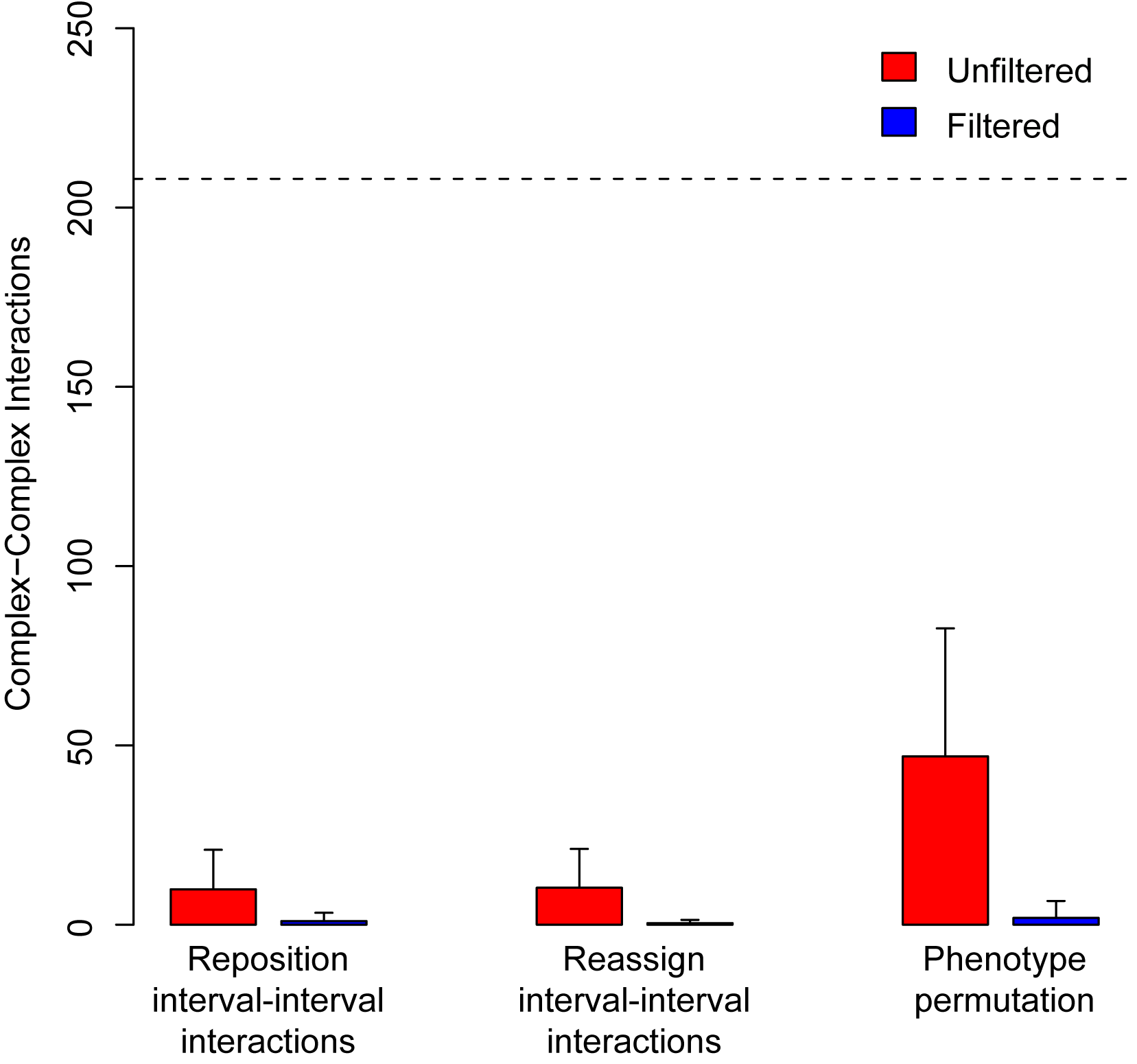

Supplement: Figure S5 — Additional permutation methods for pathway validation. The number of complex-complex interactions identified by the natural network (dotted line) is compared to the average number of complex-complex interactions identified across 100 permuted interval networks generated using three different procedures. Complex-complex interactions were mapped using either all complexes (unfiltered) or only those with a co-localization p-value above 0.1 (filtered). Error bars indicate one standard deviation. (0.29 MB TIF) [file pgen.1000782.s005.tif]
