# Supplementary material for: Genome-Wide Association Data Reveal a Global Map of Genetic Interactions among Protein Complexes
Source: PLoS Genet. 2009 Dec 24;5(12):e1000782. doi: 10.1371/journal.pgen.1000782 (PMC2788232; doi:10.1371/journal.pgen.1000782)
Supplement: Text S1 — Supplementary methods. (0.06 MB DOC) [file pgen.1000782.s014.doc]

**Supplementary Methods**

**Mapping interacting marker pairs using an exhaustive 2D scan**

In order to compare to the Storey *et al.* [1] approach, we performed a complete 2D scan of the Brem et al. [2] linkage data to identify interacting marker pairs. First, redundant markers were merged in a manner identical to Storey et al. (see Table S1 for a list of all markers and genomic positions). Next, for each gene-expression trait marker-pairs were assigned a baseline F-score for interaction using a two-way analysis of variance with a fixed-effects model [3]. To estimate significance, the complete scan was repeated over 100 permutations in which each segregant strain was randomly re-assigned a gene-expression value. The best F-score for each trait in each permutation was used to construct an empirical null distribution. This distribution was subsequently used to assign a p-value to each marker pair. For comparison with the Storey *et al.* [1] marker-pair list, marker pairs across all traits were pooled together and thresholded at P < 0.18 which produced an identical number of marker pairs (4,687; Table S7A). This network was bi-clustered (Table S7B), and examined for annotation enrichment (Table 1 in the main text). It performed substantially worse than the network derived from Storey et al.

**Annotation datasets**

The following annotation datasets were used in this study:

1. GO terms: We obtained gene functional annotations from the Gene Ontology (GO) Database revision 5.814 (July, 2008) [4].

2. Physical complexes: A set of yeast protein complexes was obtained from MIPS [5] and from Gavin *et al.* [6] (“Core” Set). The union of these sets was filtered to ensure no two complexes shared a Jaccard score (intersection / union) greater than 0.1. When two complexes exceeded this threshold, priority was given to MIPS literature-curated complexes followed by complexes with greater numbers of proteins. A list of all complexes used can be found in Table S8.

**Defining the marker pair test spaces**

Two sets of interacting marker pairs were considered in this study: (1) marker pairs from the Storey *et al.* dataset and (2) marker pairs identified through an exhaustive 2D scan. For the Storey *et al.* dataset, a marker pair was defined as tested if it had been examined for joint linkage with at least one trait. This produced 691,039 tested marker pairs. For the exhaustive 2D scan, we discarded all marker pairs which were highly correlated in the segregant population as correlated marker pairs violate the assumption of balance in two-way ANOVA (the method used in the exhaustive 2D scan as described above). Two markers were considered highly correlated if the number of segregants with the most common pair-wise genotype was more than twice that of the least common pair-wise genotype. Using this criterion, the exhaustive scan tested a total of 623,073 marker pairs (representing approximately 85% of all pairs).

**Defining the gene pair test spaces**

To determine the significance of enrichment of genetic interactions within or between annotations, we determined four parameters for the hypergeometric distribution *k*, *m*, *n*, and *N* as described in the main Methods. To compute these values, it was first necessary to determine the set of gene pairs tested by each genetic network. This space was computed differently depending on the network type, consisting of either (1) raw marker-marker interactions, (2) interval-interval interactions, or (3) synthetic gene-gene interactions. A test space (4) was also constructed for the physical complexes and functional terms.

A gene pair (g1, g2) was considered “tested” iff:

Case (1): There exists a *tested* marker pair (m1, m2) such that m1 🡪 g1 and m2 🡪 g2 (the arrows 🡪 denote mapping of markers to genes as described in the main Methods; the definition of tested marker pairs is given in the section above).

Case (2): There exists *any* marker pair (tested or untested) such that m1 🡪 g1 and m2 🡪 g2. The rationale is that an interval covers a contiguous range of markers, regardless of whether any individual marker was explicitly tested for interaction.

Case (3): The corresponding double mutant had been created and examined as part of a synthetic screen, regardless of the growth rate of the mutant. This information is not reported in every genetic interaction study but was available for the four included in our paper.

Case (4): The physical complex and functional term sets were each assigned a test space consisting of all pair-wise interactions between genes annotated in each set.

All four parameters of the hypergeometric distribution were considered only within the subset of *N*, the intersection of the test space of the analyzed network and the test space of the complexes/terms.

**Defining a colocalization score and determining a suitable threshold**

For each annotation A, a co-localization score *P_min_* was computed as follows.

Define:

G = {*g_1_*, *g_2_*, …, *g_|G|_*}, the set of genes in annotation A. Define a partition G_c_$\subseteq$G which contains all *g* in G that fall on chromosome *c*.

**x** = (*x_1_*, *x*_2_, …, *x_|G|_*), the genomic position of each *g* in G on its chromosome, measured in bp from the left chromosome end to the middle of the ORF encoding *g* (as given in the SGD database [7]).

**m** = (*m_1_,* *m_2_, …, m_16_*), the co-localization score to be determined for each chromosome.

For each chromosome *c*  = (1,2, …16):

For all (*g_i_*, *g_j_*) in G_c_, compute the intergene distance *d_ij_* = |*x_i_* – *x_j_*|.

Define **d_c_** = (*d_1_*^*^, *d_2_*^*^, …) as the sorted list of these intergenic distances.

Compare each *d_i_*^*^ to a corresponding null distribution of distances **d_i_^0^**. **d_i_^0^** is the distribution of gene-gene distances at rank *i* in the sorted list produced by sampling without replacement the same number of genes |G_c_| at random from the chromosome 10^6^ times. Define **p =** (*p_1_,p_2_, …*) where *p_i_*is the p-value of *d_i_^*^* indexed against **d_i_^0^.**

*m_c_* = min(**p**).

*P_min_* = min(**m**)

Given this metric, *P_min_*, co-clustered annotations were filtered by removing those annotations with *P_min_* < *p_T_* from further consideration. We chose a suitable threshold *p_T_* so as to ensure that no complex-complex or term-term associations would be reported given a permuted network. Permuted interval networks were generated by randomly assigning a new starting marker-index to each interval in the natural network, while ensuring that interval pairs remain disjoint and that no interval crosses the edge of a chromosome. We computed the number of significant associations found by 100 permuted networks over a range of *p_T_* values (Figure S4). Based on this analysis, we chose a stringent colocalization threshold of *P_min_*>0.1 for physical complexes and *P_min_*>0.3 for functional terms (blue arrows in Figure S4) resulting in less than one erroneous complex-complex or term-term interaction identified per permuted network.

Physical complexes and terms with a score above these thresholds were removed prior to enrichment analysis. The filtering process removed approximately 16% of the physical complexes and 40% of the functional terms, resulting in a reduced set of 302 physical complexes (Table S8) and 1,954 functional terms after further processing as described above.

To further validate the annotation models, we performed two additional permutation methods and examined how many significant complex-complex interactions could be identified in either case. First, we re-assigned interactions between intervals in the natural network. Second, we performed 100 randomized scans for marker-marker interactions using the method of Storey *et al*. [1], in which the associations between segregant strains and gene expression traits were randomly permuted while leaving the associations between segregrant strains and genotypes intact. Each of these random marker-marker networks was subsequently bi-clustered to produce interval-interval networks. Across, 100 permuted interval networks derived through these two methods very few complex-complex interactions were identified (<2 on average; Figure S5).

**Determining significance of overlap between natural and synthetic networks**

The overlap between the natural and synthetic networks was based on the number of synthetic genetic interactions that were supported by the natural network. A genetic interaction was considered supported if the two genes mapped into two different genomic intervals that were found to interact. Significance was determined using 1,000 natural network permutations using a procedure based on the re-positioning of interval-interval interactions. In this scheme, each interval of an interval-interval interaction in the natural network was randomly assigned a new starting marker-index, while ensuring that interval pairs remain disjoint and that no interval crosses the edge of a chromosome. This effectively disrupts any biological signal, while preserving the distribution of interval sizes.

**Mapping broad GO terms**

As shown in Figure 3B of the main text, we characterized the functional relationships for the natural and synthetic networks by mapping all identified functional term and term-term interactions to a set of “broad” terms defined at the fifth and sixth levels of the GO hierarchy (1,285 possible terms). For each of these broad terms, the number of term and term-term interactions among the mapped children was tabulated. Similarly, for each pair of broad terms, the number of term-term interactions between the respective children was tabulated. The 10 broad terms and 30 term-term interactions with the most counts were considered a good representation of the functional relationships evident in the natural and synthetic networks.

**Supplementary References**

1. Storey J, Akey J, Kruglyak L (2005) Multiple locus linkage analysis of genomewide expression in yeast. PLoS Biol 3: e267.

2. Brem R, Storey J, Whittle J, Kruglyak L (2005) Genetic interactions between polymorphisms that affect gene expression in yeast. Nature 436: 701-703.

3. Sahai H, Ageel MI (2000) The analysis of variance : fixed, random, and mixed models. Boston: Birkhäuser. xxxv, 742 p.

4. Ashburner M, Ball C, Blake J, Botstein D, Butler H, et al. (2000) Gene ontology: tool for the unification of biology. The Gene Ontology Consortium. Nat Genet 25: 25-29.

5. Mewes H, Frishman D, Güldener U, Mannhaupt G, Mayer K, et al. (2002) MIPS: a database for genomes and protein sequences. Nucleic Acids Res 30: 31-34.

6. Gavin A, Aloy P, Grandi P, Krause R, Boesche M, et al. (2006) Proteome survey reveals modularity of the yeast cell machinery. Nature 440: 631-636.

7. Cherry J, Ball C, Weng S, Juvik G, Schmidt R, et al. (1997) Genetic and physical maps of Saccharomyces cerevisiae. Nature 387: 67-73.
